# Maternal Latent *Mycobacterium tuberculosis* Does Not Affect the Infant Immune Response Following BCG at Birth: An Observational Longitudinal Study in Uganda

**DOI:** 10.3389/fimmu.2020.00929

**Published:** 2020-05-14

**Authors:** Lawrence Lubyayi, Patrice A. Mawa, Grace Nabakooza, Marjorie Nakibuule, John Vianney Tushabe, Joel Serubanja, Dorothy Aibo, Hellen Akurut, Josephine Tumusiime, Mateusz Hasso-Agopsowicz, Pontiano Kaleebu, Jonathan Levin, Hazel M. Dockrell, Steven Smith, Emily L. Webb, Alison M. Elliott, Stephen Cose

**Affiliations:** ^1^Immunomodulation and Vaccines Programme, Medical Research Council/Uganda Virus Research Institute and London School of Hygiene and Tropical Medicine Uganda Research Unit Entebbe, Entebbe, Uganda; ^2^Department of Epidemiology and Biostatistics, School of Public Health, University of the Witwatersrand, Johannesburg, South Africa; ^3^Department of Immunology, Uganda Virus Research Institute, Entebbe, Uganda; ^4^Department of Infection Biology, London School of Hygiene and Tropical Medicine, London, United Kingdom; ^5^Department of Life Sciences, Brunel University London, London, United Kingdom; ^6^MRC Tropical Epidemiology Group, Department of Infectious Disease Epidemiology, London School of Hygiene and Tropical Medicine, London, United Kingdom; ^7^Department of Clinical Research, London School of Hygiene and Tropical Medicine, London, United Kingdom

**Keywords:** latent *Mycobacterium tuberculosis* infection, maternal infection, BCG vaccine, cytokine responses, antibody responses

## Abstract

**Background:** BCG has low efficacy in tropical countries. We hypothesized that maternal latent *Mycobacterium tuberculosis (M.tb)* infection (LTBI) results in fetal tolerance to mycobacterial antigens and impaired responses to BCG immunization.

**Methods:** We enrolled 132 LTBI-positive and 150 LTBI-negative mothers and their babies in Entebbe, Uganda. Infants were BCG-immunized at birth. Cord blood and samples at weeks 1, 4, 6, 10, 14, 24, and 52 were analyzed for cytokine/chemokine responses to *M.tb* antigens by Luminex 17-plex assay in 6-day whole blood cultures and antibody responses by ELISA. Of the 17 Luminex analytes, seven (IL-2, IL-5, IL-10, IL-13, IL-17A, TNF, and IFN-γ) were included in the main analysis as they were considered most likely to represent T cell responses. Immune sensitization was defined as a detectable cord blood cytokine response to PPD for any of the seven cytokines. Patterns of cytokine and antibody responses were compared between infants of mothers with and without LTBI using linear mixed models adjusting for confounders.

**Results:** Most infants (73%) were sensitized *in utero* to *M.tb* antigens, with no overall difference seen between infants born to mothers with or without LTBI. Patterns of post-BCG cytokine and antibody responses to mycobacterial antigens were similar between the two infant groups.

**Conclusions:** Our data do not support the hypothesis that maternal LTBI results in an impaired response to BCG immunization, in Ugandan infants. BCG vaccination at or shortly after birth is likely to be beneficial to all infants, irrespective of maternal LTBI status.

## Introduction

Bacille Calmette-Guerin (BCG) is the only licensed vaccine against tuberculosis (TB). It protects against tuberculous meningitis and miliary TB in infants (1), but its protective efficacy against pulmonary TB varies between populations. Meta-analyses of BCG vaccine trials have shown that latitude is an important factor for responses in adolescents and adults, with lower protection closer to the equator (2–5).

Modification of the protective effect of BCG through sensitization to non-tuberculous mycobacteria (NTMs) has been suggested as a reason for variable BCG efficacy, and its association with latitude (6, 7). The protective effects of BCG might be blocked by exposure to NTMs, or NTMs might provide equivalent protection to BCG, thus masking the benefit provided by BCG (8). Although NTMs have a variable distribution by latitude (9), NTM exposure may not fully explain this variability (10).

In TB endemic areas, BCG is administered to new-borns at birth, in accordance with WHO recommendations (11). BCG elicits different profiles of immune response in Africa compared with the UK when given early in life (12). Prior sensitization, perhaps due to early exposure to *Mycobacterium tuberculosis (M.tb)* itself, or to environmental mycobacteria has been reported in infants immunized some months after birth (13). However, *in utero*, rather than early life, sensitization (14) may result in a more substantial modification of responses in exposed infants.

In TB endemic areas, a high proportion of adults harbor latent *M.tb* infections (LTBI). A dynamic relationship between mycobacteria and the immune system is thought to exist during LTBI. Individuals with LTBI may have circulating antigens and higher concentrations of TB-specific antibodies, plasmablasts, and memory B cells than those without infection (15, 16). Mycobacterial antigens cross the placenta in murine models (17). Thus, maternal LTBI might lead to exposure to mycobacterial antigens *in utero* driving a modified profile of sensitization (18), or inducing tolerance in the fetus (14, 19). Alternatively, passive transfer of maternal anti-mycobacterial antibodies (by providing passive immunity) or maternal anti-idiotype antibodies (mimicking antigen) (20), might influence the ability of neonatal BCG vaccine to elicit protective immune responses. The maternal and placental immunological milieu could also be influenced non-specifically by maternal LTBI, with consequences for fetal and neonatal response following immunization (21). For other pathogens, maternal infections have been shown to induce either tolerization or sensitization in the fetus, with subsequent differences in susceptibility to infection (22). We previously showed impaired mycobacteria-specific T-cell responses following BCG immunization of infants born to LTBI-positive mothers, although this effect appeared to be transient (23).

We hypothesized that maternal LTBI influences the neonatal response to mycobacteria, impairing the response to BCG and *M.tb*. To investigate this we followed a cohort of infants of LTBI infected or uninfected mothers over the first year of life. We measured cellular responses induced by neonatal BCG immunization using a whole blood assay (24). As well, the evolution of anti-mycobacterial antibody responses was assessed, since these have recently gained new recognition for a potential role in protective immunity against tuberculosis (25–28). We measured responses to both the relatively *M.tb*-specific antigens (ESAT6 and CFP10) and to the broadly mycobacterial-specific purified protein derivative (PPD) in order to evaluate exposure to *M.tb*, and acquisition of responses to *M.tb, in utero* and during the first year of life, as distinct from the response to BCG.

## Materials and Methods

### Study Design and Participants

Healthy mothers and their infants were recruited at Entebbe General Hospital between June 2014 and October 2016. Women who were willing to participate in the study, had a normal singleton pregnancy, resided in Entebbe municipality or neighboring Katabi sub-county, and were HIV negative were eligible for inclusion. They were excluded if cord blood was not obtained, delivery was not normal, the mother was unwilling to undergo a repeat HIV test or was found to be HIV-positive on repeat testing, birth weight was <2,500 g, the neonate was unwell as judged by the midwife, the mother had indeterminate LTBI status (as described below), or the neonate presented with significant congenital abnormalities likely to impair the child's general health and development. Enrolled infants received all vaccines recommended by the Expanded Programme on Immunization. Infants were included in the study based on their mother's LTBI status, targeting equal numbers of infected and uninfected women. All infants were immunized at birth or within the first week of life with a single dose of intradermal BCG (Statens Serum Institut (SSI), Denmark).

### Blood Sampling Strategy

Up to 7 ml of cord blood was collected. Infants were then randomly assigned in a 1:1 ratio (stratified by LTBI status) to two sampling strategies to reduce the blood-sampling burden on individual infants. Half gave 2 ml venous blood at 1, 6, and 14 weeks, the remainder at 4, 10, and 24 weeks. All gave blood (5 ml) at 52 weeks. Blood draws at weeks 14 and 24 were introduced when the study was already underway, resulting in lower sample numbers at these times.

### Tests for Latent TB Infection

Women were investigated for LTBI at approximately 1 week post-delivery using the tuberculin skin test (TST) (PPD RT23 SSI, Copenhagen, Denmark) and T-SPOT.TB assay (Oxford Immunotec, Abingdon, UK) (29). The TST was performed in the mothers after bleeding for the T-SPOT.TB assay and was read 48–72 h later, and defined as positive if ≥10 mm in diameter. Women positive on both tests were considered LTBI-positive; those negative on both tests were considered LTBI-negative. Those with indeterminate and discordant results were excluded in order to optimize our ability to determine effects of LTBI (as distinct from other mycobacterial exposures). LTBI-positive mothers were investigated for active tuberculosis by symptoms, sputum examination (if available), and chest x-ray. No cases of active TB were detected.

### Whole Blood Assays for Cytokine/Chemokine Responses

Whole blood (including cord and infant venous blood) was diluted 1 in 5 in RPMI 1640 (Invitrogen) supplemented with 2 mM L-glutamine (Invitrogen) and cultured under 5% carbon dioxide at 37°C for 6 days in 96-well U-bottomed plates (final volume 200 μl). Duplicate wells were incubated with medium alone (negative control), PPD (Statens Serum Institut, catalog #RT50) (10 μg/ml), or a combination of ESAT6 and CFP10 antigens (BEI Resources, calatologue #sNR14868 and NR-49425) (5 μg/ml).

After 6 days, plates were centrifuged at 400 g for 5 min. Supernatants were removed from duplicate wells, pooled, and stored at −80°C prior to analysis. Thawed supernatants were randomized across plates and subjected to multiplex bead array analysis using the human cytokine/chemokine MilliplexTM MAP 17-plex pre-mixed kit (Merck Millipore), following the manufacturer's instructions. The pre-mixed bead set included interleukin (IL)-1α, IL-1β, IL-1Ra, IL-2, IL-5, IL-8, IL-10, IL-12p40, IL-13, IL-17A, interferon (IFN)-γ, IFN-γ-inducible protein (IP)-10, monocyte chemotactic protein (MCP)-1, macrophage inflammatory protein (MIP)-1α, MIP-1β, tumor necrosis factor (TNF), and granulocyte macrophage colony-stimulating factor (GM-CSF). Data were acquired using the Biorad Luminex® 200 system and Bioplex Manager Software version 6.1 (Biorad).

### ELISA for Anti-mycobacterial IgG Antibodies

Total immunoglobulin (Ig)G against PPD, ESAT6, CFP10, and Ag85A were assayed in plasma of a random sample of infants, at each time point, by ELISA as described elsewhere (23). Briefly, flat-bottomed 96-well microlon plates (Greiner Bio-one, Germany) were coated with purified IgG standard (GenScript, NJ, USA) and mycobacterial antigens. After overnight incubation, the plates were blocked and samples diluted 1 in 100 were added to the plates and left overnight at 4°C. Polyclonal anti-human IgG Horse Radish Peroxidase (Poly HRP, 0.5 μg/ml, Dako, Denmark) was added and plates incubated. 0-Phenylenediamine (OPD, Sigma-Aldrich, MO, USA) substrate mixture (3 mg OPD, 0.1M citric acid, 0.2M Na2HPO4, 3 μL 30% hydrogen peroxide in distilled water) was then added and the reaction stopped with 2M Sulphuric acid and read at test wavelength 490 nm and reference wavelength 630 nm using a MRX1.1 plate reader and Gen5 1.07 Software (BioTek Instruments, Inc., VT, USA). The lowest standard concentration above which antibody was detectable (0.01 μg/ml) was set as the sensitivity of the assay.

### Statistical Methods

Analyses studied the time course of PPD- and ESAT6/CFP10-specific responses at 1, 4, 6, 10, 14, 24, and 52 weeks after BCG immunization, and the influence of maternal LTBI on infant responses.

We aimed to recruit 150 women with LTBI and 150 without, to give 80% power to detect a difference of 0.35log10 (assuming a standard deviation of 0.9log10) (30) in infant cytokine response at 52 weeks between the two groups, and a difference of 0.5log10 at other time points (with 75 infants in each group).

Unstimulated cytokine response values were subtracted from antigen-stimulated results. Values <3.2 pg/mL (lower detection limit of assay) were assigned as 3.2 pg/mL. Values above 11,000 pg/mL (the upper detection limit) were assigned 11,000 pg/mL.

Baseline characteristics of participants were summarized, by LTBI status, using percentages, means and standard deviations, and medians and interquartile ranges. Of the 17 Luminex analytes, seven (IL-2, IL-5, IL-10, IL-13, IL-17A, TNF, and IFN-γ) were included in the main analysis as they were considered most likely to represent T cell responses. Results from the remaining ten cytokines were included in supplementary analyses.

Principal components analysis (PCA), a procedure which transforms several (possibly) correlated variables into a smaller number of uncorrelated variables (principal components), was used on cord blood outcomes to investigate relationships between the seven cytokine responses. We defined immune sensitization as a detectable cytokine response to PPD, in cord blood, for any of the seven cytokines. We summarized proportions of infants sensitized based on each of the seven cytokines and on groups discovered by PCA, and compared these proportions by maternal LTBI status using chi-squared tests.

Changes in log-transformed cytokine responses over time, by mother's LTBI status, were studied using linear mixed models adjusted for factors that showed baseline differences between the two groups. Profile plots showing the mean concentration and 95% confidence intervals (CI) were used to visualize the differences. Similar plots were generated for antibody responses.

Three-way component analysis (31) was used to explore relations between cytokine responses, following BCG, taking into account trends over all time points.

Data analysis was conducted using Stata 14.1 (College Station, Texas, USA) and R version 3.5.1 (R Foundation for Statistical Computing, Vienna, Austria). A 5% significance level was used for all analyses.

### Ethical Approvals

Ethical approval was given by the Uganda Virus Research Institute Research Ethics Committee (reference GC/127/13/09/16 and GC/127/16/03/434), Uganda National Council for Science and Technology (reference HS 1526) and the London School of Hygiene & Tropical Medicine (reference 7104). Written informed consent was received from all mothers, for their own and their baby's participation.

## Results

### Participants' Characteristics

**Figure 1:**
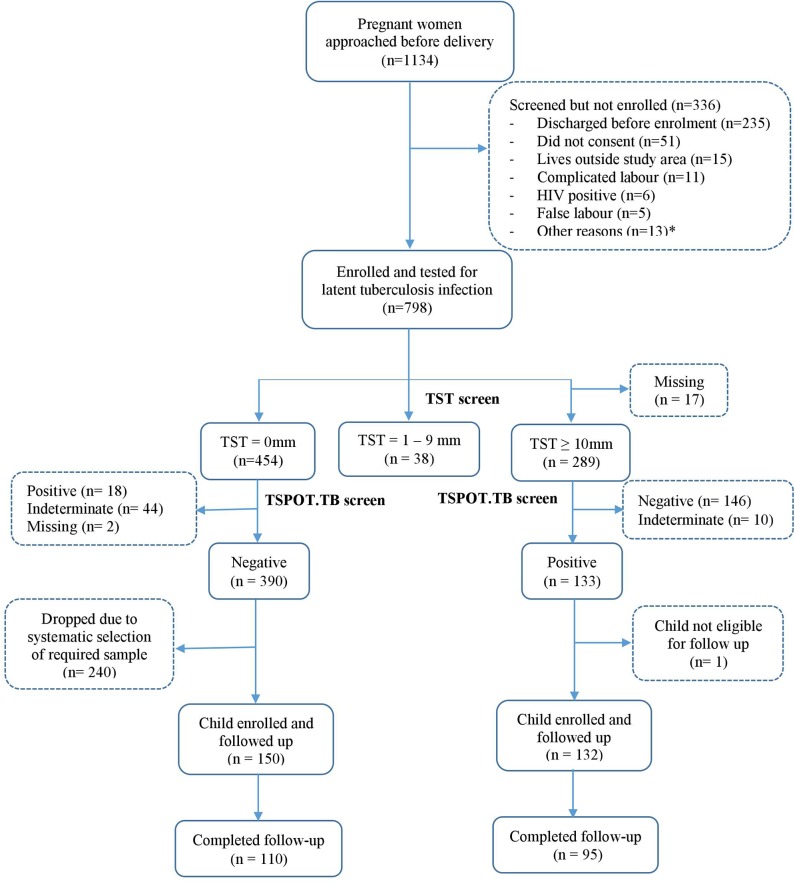
Study flowchart. *Other reasons include discharged before consenting procedures, low birthweight babies, cord blood not taken, asphyxia, child not vaccinated, and birth to twins.

The study flowchart is shown in Figure 1. Between June 2014 and October 2016, 1134 women were invited to participate, 798 (70%) of whom consented and were tested for LTBI. 390 women tested negative on both TST and TSPOT.TB while 133 were positive on both tests. Among the 390 LTBI-negative women, systematic sampling was done to randomly select those for enrolment: initially we enrolled every second woman, later every third woman, to ensure contemporaneous recruitment with LTBI-positive women. Of the 133 LTBI-positive women, 132, with their infants, were eligible for follow up and enrolled into the study.

Baseline characteristics of participants were similar between the two groups (Table 1), except that LTBI-positive mothers were on average older, more likely to have lived with someone who had TB, to drink alcohol and to originate from the central region of Uganda. Infant characteristics (sex and birth weight) were similar between the two groups.

**Table 1:** Characteristics of study participants.

	**LTBI Negative**		**LTBI Positive**	
	**(*****n*** **=** **150)**		**(*****n*** **=** **132)**	
**Maternal characteristics**				
Mean mother's age (SD) (mv 0, 3)^a^	23.65	(3.67)	25.53	(4.99)
Median number of pregnancies (IQR) (mv 1, 1)	2	(1-3)	2	(2-4)
Median number of births (IQR) (mv 1, 1)	2	(1-3)	2	(1-3)
Positive malaria test during pregnancy (mv 1, 1)	38	25.5%	29	22.1%
Ever lived with someone with TB (mv 2, 1)	3	2.0%	19	14.5%
BCG scarring (mv 1, 2)	106	71.1%	95	73.1%
Ever taken any medicine for worms (mv 1, 2)	135	90.6%	116	89.2%
**Current marital status (mv 2, 4)**				
Single	28	18.9%	20	15.6%
Married/living as married	120	81.1%	108	84.4%
**Highest level of education attained (mv 2, 3)**				
Never attended school	5	3.4%	2	1.5%
Primary	50	33.8%	44	33.9%
Secondary	81	54.7%	66	50.8%
Tertiary	12	8.1%	17	13.1%
Smoked in the past (mv 1, 1)	1	0.7%	1	0.8%
Drink alcohol (mv 3, 4)	18	12.2%	28	21.9%
*Schistosoma mansoni* infected (mv 33, 36)	13	11.1%	9	9.4%
Hookworm infected (mv 33, 36)	9	7.7%	2	2.1%
*Trichuris* infected (mv 33, 36)	1	0.9%	1	1.0%
Any helminth infection (mv 33, 36)	20	17.1%	12	12.5%
**Mother's tribe grouping (mv 5, 3)**				
Central	56	38.6%	72	55.8%
Other	89	61.4%	57	44.2%
**Father's tribe grouping (mv 3, 2)**				
Central	58	39.5%	75	57.7%
Other	89	60.5%	55	42.3%
**Infant characteristics**				
Sex of the baby, male	77	51.3%	77	58.3%
Mean birth weight in kg (SD)	3.24	(0.43)	3.21	(0.40)

*Data are mean (SD), median (IQR), or n (%). SD, standard deviation; IQR, interquartile range; mv, missing values*.

a *Figures in parentheses indicate missing values in the LTBI-Negative and LTBI-Positive groups, respectively*.

Due to the study design, not all infants provided samples at all-time points. Supplementary Table 1 shows sample numbers assayed at each time point.

### Cord Blood Outcomes and Immune Sensitization

Based on cord blood responses to PPD for any of the seven cytokines IL-2, IL-5, IL-10, IL-13, IL-17A, TNF, and IFN-γ, 73% of the infants made a positive cytokine response to *M.tb* antigens, with no overall difference seen between infants born to mothers with or without LTBI (78 vs. 69%, respectively; *p* = 0.119). Considering individual cytokines, a larger proportion of infants born of LTBI-positive mothers had PPD-specific TNF responses in cord blood as compared to those born of LTBI-negative mothers (74 vs. 61%, respectively; *p* = 0.038) (Table 2).

**Table 2:** Immune sensitization based on cord blood responses to PPD.

	**LTBI-Negative**		**LTBI-Positive**		
**Cytokines**	**(*****n*** **=** **138)**		**(*****n*** **=** **116)**		***p*-value**
**Individual cytokines**					
IL2	12	8.7%	10	8.6%	0.983
IL5	7	5.1%	7	6.0%	0.738
IL10	38	27.5%	45	38.8%	0.057
IL13	21	15.2%	17	14.7%	0.900
IL17A	13	9.4%	7	6.0%	0.318
TNF	84	61.3%	84	73.7%	0.038
IFN-γ	17	12.3%	14	12.1%	0.952
**Based on any of the 7 cytokines**					
IL2, IL5, IL10, IL13, IL17A, TNF and IFN-γ	95	68.8%	90	77.6%	0.119
**Based on PCA grouping**					
IL2, IL5, IL13, IL17A and IFN-γ	30	21.7%	26	22.4%	0.897

For ESAT6/CFP10 responses, there was no overall difference in responses between infant groups, and >90% of all infants had a positive TNF response (Supplementary Table 2).

**Figure 2:**
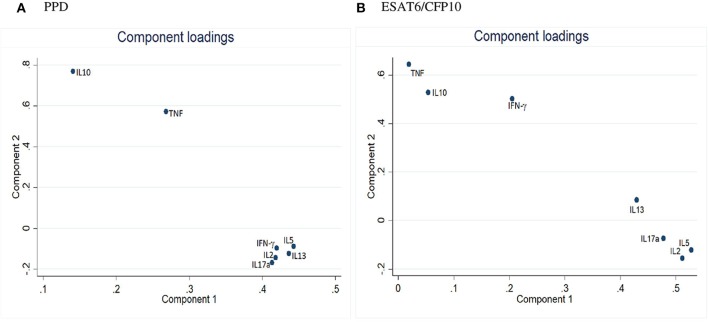
Principal Component Analysis of cord blood outcomes in Ugandan infants. **(A)** PCA for cord blood cytokine responses to PPD. **(B)** PCA for cord blood cytokine responses to ESAT6/CFP10.

PCA component loadings for cord blood cytokine responses to both PPD and ESAT6/CFP10, are shown in Figure 2. The cytokine responses to PPD, IL-10 and TNF clustered separately from the other cytokines. The cytokine responses to ESAT6/CFP10, IL-10, TNF, and IFN-γ clustered separately from the other cytokines. The PCA clustering profiles described did not differ between the two infant groups.

### Maternal LTBI and Infant Cytokine Responses Over Time

**Figure 3:**
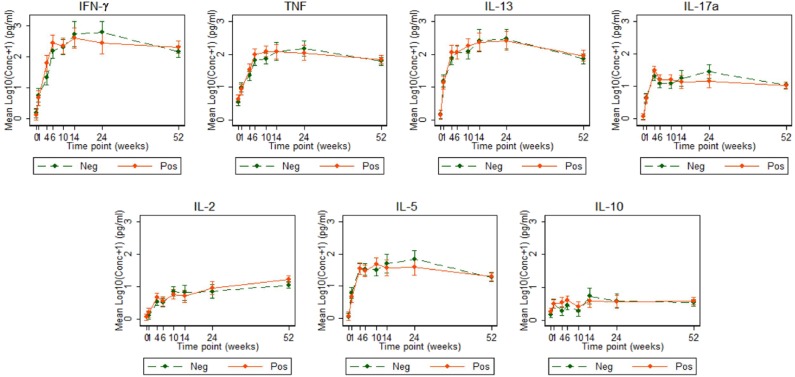
Cytokine responses to PPD in Ugandan infants born of mothers with or without LTBI. PPD-specific cytokine concentrations corrected for background (on the log scale). Points represent mean values and the bars represent 95% confidence intervals around the mean. The solid and dashed lines represent concentrations from children born of LTBI-positive and LTBI-negative mothers, respectively.

**Figure 4:**
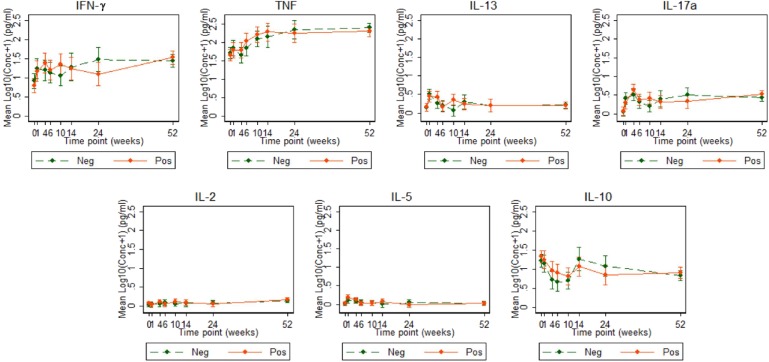
Cytokine responses to ESAT6/CFP10 in Ugandan infants born of mothers with or without LTBI. ESAT6/CFP10-specific cytokine concentrations corrected for background (on the log scale). Points represent mean values and the bars represent the 95% confidence intervals around the mean. The solid and dashed lines represent concentrations from children born of LTBI-positive and LTBI-negative mothers, respectively.

**Figure 5:**
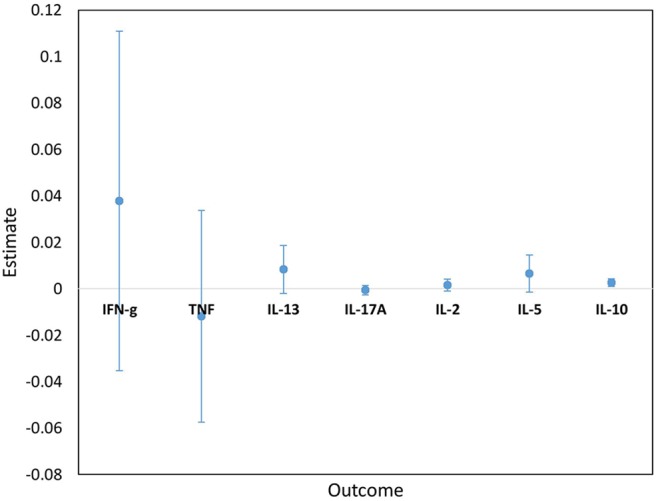
Estimates for the interaction terms between time and LTBI status for cytokine responses to PPD. Estimates and 95% confidence intervals for the interaction term between time and LTBI from univariate linear mixed models for cytokine responses to PPD.

**Figure 6:**
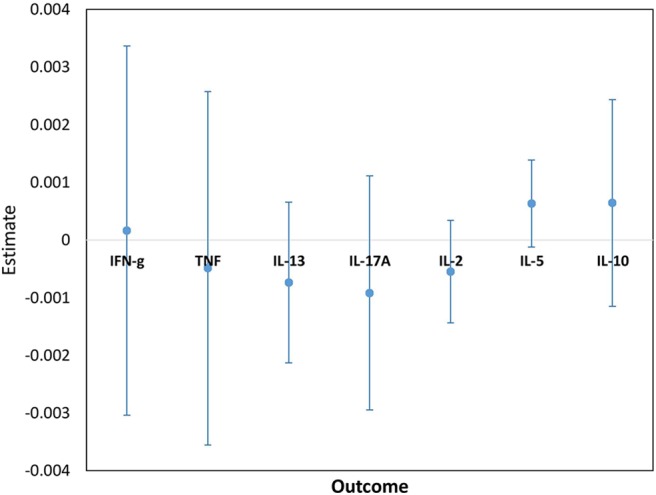
Estimates for the interaction terms between time and LTBI status for cytokine responses to ESAT6/CFP10. Estimates and 95% confidence intervals for the interaction term between time and LTBI from univariate linear mixed models for cytokine responses to ESAT6/CFP10.

Cytokine responses to both PPD and ESAT6/CFP10 were similar, at all-time points, between the two infant groups (Figures 3, 4 and Supplementary Table 3). The peak of the infant response to PPD was sustained from 10 to 24 weeks of age (Figure 3). From univariate linear mixed models, adjusted for sex of the infant and factors that showed baseline differences between the two groups (mother's age, household TB contact, alcohol consumption, parental tribe), there was no evidence of interaction between time and mother's LTBI status for any of the seven outcomes for either PPD or ESAT6/CFP10 responses (Figures 5, 6), demonstrating that the association between LTBI status and the responses did not change over time.

Cytokine responses to ESAT6/CFP10 varied over time. IFN-γ and TNF responses started high, increased up to about 24 weeks and then plateaued; IL-13 and IL-17A responses increased up to 4 weeks, declined up to 10 weeks and then plateaued; IL-2 and IL-5 responses were consistently very low, whilst IL-10 responses declined up to 10 weeks, increased up to 14 weeks and then plateaued (Figure 4). These responses also showed no difference between the groups over time.

PPD and ESAT6/CFP10 responses for the other 10 cytokines/chemokines included in the array (IL-8, MCP-1, MIP-1α, MIP-1β, IL-1α, IL-1β, IL-12, IL-1Ra, GM-CSF, and IP-10) were also similar between the two infant groups (Supplementary Figures 1, 2).

### Cord Blood Sensitisation and Infant Cytokine Responses Over Time

There was no difference in evolution of PPD responses based on cord blood response profiles (Supplementary Figure 3).

### Antibody Responses Over Time

**Figure 7:**
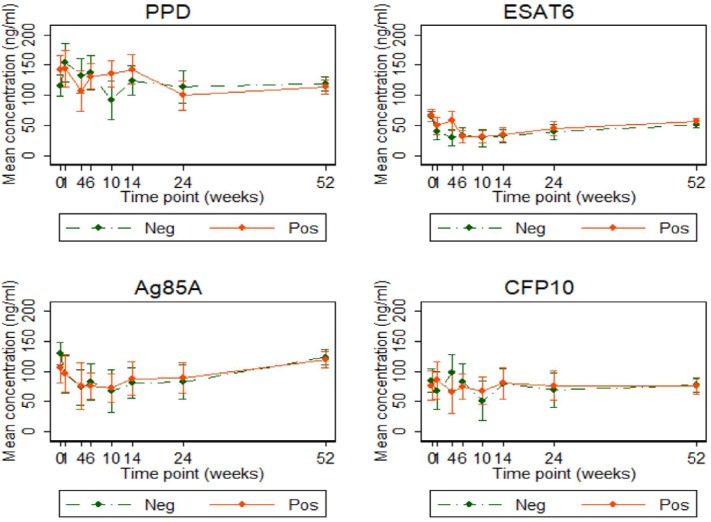
Antibody responses to PPD, ESAT6, Ag85A, and CFP10 in infants born of mothers with or without LTBI. Antibody responses to PPD, ESAT6, Ag85A, and CFP10 (ng/ml). Points represent mean values and the bars represent 95% confidence intervals around the mean. The solid and dashed lines represent concentrations from children born of LTBI-positive and LTBI-negative mothers, respectively.

Antibody concentrations were similar, at all-time points, between the two infant groups. Anti-ESAT6 and anti-Ag85A IgG antibody concentrations declined up to 10 weeks and then gradually increased, consistent with maternal antibodies (in both groups) waning and then infants generating their own antibodies. There was no difference in antibody concentrations over time, based on cord blood response profiles. Antibody levels to PPD, Ag85A, and CFP10 were generally higher than those to ESAT6 (Figure 7).

### Associations Between Cytokine Response Dynamics Over Time

Results from three-way component analyses show that IL-10, IL-2, and IL-5 responses to PPD evolved in a similar way (Supplementary Figure 4A), and TNF and IL-17 responses evolved similarly to each other, whereas IL-13 and IFN-γ responses separated out independently from the other cytokines. Responses to ESAT6/CFP10 clustered for IL-13, IL-5, and IL-17 (Supplementary Figure 4B), indicating similar evolution patterns over time; TNF, IL-10, IFN-γ, and IL-2 were separated from the other cytokines indicating unique evolution patterns over time. However, there were no differences in clustering patterns between the groups based on mother's LTBI status.

## Discussion

In this study, we show that Ugandan infants were sensitized to mycobacterial antigens *in utero*, regardless of maternal LTBI status, and that maternal LTBI had no effect on evolution of immune responses to BCG over time.

Maternal infections during pregnancy, with various pathogens, sensitize the fetus *in utero* (14, 18, 32, 33), and prenatal *M.tb* sensitization has long been recognized among mothers from TB-endemic settings (14). In addition, maternal helminth co-infection appears to modify the infant response to subsequent infection with the same pathogen (22), and to BCG immunization (34). In a small preliminary study, we demonstrated impaired mycobacteria-specific T-cell responses following BCG immunization of infants born to LTBI-positive mothers, although this effect was transient (23). We therefore postulated that maternal *M.tb* infection would strongly influence fetal sensitization and the neonatal response to BCG immunization. Our new results refute this hypothesis. A previous study from South Africa showed that maternal HIV infection had an effect on infant immune responses in the presence of maternal *M.tb* sensitization at birth, however, these effects were not maintained post immunization with BCG (35). Our study design differed from the South African study in terms of earlier BCG immunization (at birth, rather than age 6 weeks), larger sample size, exclusion of HIV positive mothers, use of two tests (TST and T-SPOT.TB) rather than one (QuantiFERON-TB Gold In-Tube) to rigorously distinguish LTBI-positive or negative mothers, and more frequent sampling during infancy. Together, the two studies provide clear evidence that maternal LTBI does not impact the infant response to BCG in endemic settings.

PCA in cord blood showed IL-10 and TNF tending to group separately from the other five cytokine responses, but there was no evidence that maternal LTBI was associated with a differing pattern of response, or that differences in these cord blood profiles impacted the subsequent infant response to BCG.

This conclusion is surprising in view of the recognized effects of prenatal exposure to other pathogens on neonatal immune responses. One possible explanation lies in the observation that, in our study, a very high proportion of infants demonstrated cord blood cytokine responses to mycobacterial antigens, regardless of maternal LTBI status. The mechanism of fetal sensitization may involve transfer of either antigen or antibody. Interestingly, antibody to PPD and to ESAT6/CFP10 was found in the cord blood of almost all our study infants, in considerable concentrations, implying that exposure to mycobacteria, whether NTM or *M.tb*, was almost universal. Passive transfer of antibodies appears to have occurred regardless of maternal LTBI status. Nevertheless, how fetal sensitization to mycobacterial antigen occurs in the absence of maternal LTBI needs further investigation.

A second possible explanation for the lack of difference in response between infants of mothers with and without LTBI is that the BCG stimulus is sufficiently strong to override effects of prior *in utero* exposures. Our principal component analysis of cord blood responses suggested distinct groups with IL-10 (potentially suggestive of tolerization) separate from Th1 or Th2 cytokine responses for PPD, and Th1 vs. Th2 biased groups for ESAT6/CFP10. However, these initial sensitization patterns were not reflected in the profile of response that developed following BCG. In addition to driving antigen-specific responses, it is possible that BCG-induced increases in function of innate immunity, through trained immunity, could have contributed to a lack of difference in responses between infants born of mothers with or without LTBI (36). However, additional analyses using techniques such as intracellular cytokine staining would be needed to assess the extent of cytokine production from innate cells compared to antigen-specific lymphocytes, whether T cells or B cells.

In our study, the peak infant T cell response to PPD was sustained from age 10 to 24 weeks, later than the 6–10 week period previously reported (37). This is important, as the peak for BCG-induced responses should inform future prime-boost strategies regarding timing of the boost—perhaps boosters should be given much later than has previously been thought. Our study differed from the South African study (37) in terms of the geographical location and the use of whole blood stimulation with Luminex assays to measure analytes in supernatant, as compared to flow cytometry.

Comparing UK BCG-vaccinated and un-vaccinated infants, BCG vaccination induced several cytokines and chemokines (IFN-γ, TNF, IL-2, IL-6, IL-1α, IL-4, IL-5, IL-13, IL-10, IL-8, IP-10, MIP-1α, G-CSF, and GM-CSF) (38). Our results are comparable: infants in our study produced similar cytokines, although it was not possible to determine which cells produced them.

This was the largest study of its kind to date. Although recruitment (and hence statistical power) was slightly below target, there was no suggestion of any consistent or persistent differences between the two groups. Thus, the small reduction in power is unlikely to explain the lack of differences seen. Use of a whole blood assay means that we cannot be sure about the cellular source of the analytes measured; for this analysis we have focussed on those most likely to be of T-cell origin; moreover, given that analytes were measured after 6 days of culture.

In conclusion, our data suggest remarkably high early exposure to mycobacterial antigens *in utero* in Uganda, but no impact of this exposure on the infant response to BCG. Our data do not support the hypothesis that prenatal exposure to antigens or antibodies resulting from maternal LTBI results in impaired cytokine responses induced by BCG immunization. The implication of our findings is that maternal LTBI is not the reason for reduced effectiveness of BCG in the tropics and all infants are likely to benefit from BCG immunization regardless of their maternal LTBI status.

## Data Availability Statement

The raw data supporting the conclusions of this article will be made available by the authors, without undue reservation, to any qualified researcher.

## Ethics Statement

The studies involving human participants were reviewed and approved by Uganda Virus Research Institute Research Ethics Committee (reference GC/127/13/09/16 and GC/127/16/03/434), Uganda National Council for Science and Technology (reference HS 1526) and the London School of Hygiene & Tropical Medicine (reference 7104). Written informed consent to participate in this study was provided by the participants' legal guardian/next of kin.

## Author Contributions

AE, SC, PK, EW, SS, and HD conceived the study and secured funding. AE, SC, SS, HD, EW, LL, and PM reviewed the data and wrote the initial drafts of the manuscript. LL, EW, and JL provided statistical expertise and support. PM, GN, MN, JTus, and MH-A performed all laboratory assays. JS, DA, HA, and JTum were members of the clinical team involved in recruitment, follow up and clinical reporting. All authors read and approved the final version of the manuscript.

## Conflict of Interest

The authors declare that the research was conducted in the absence of any commercial or financial relationships that could be construed as a potential conflict of interest.
