# Supplementary material for: Maternal Latent Mycobacterium tuberculosis Does Not Affect the Infant Immune Response Following BCG at Birth: An Observational Longitudinal Study in Uganda
Source: Front Immunol. 2020 May 14;11:929. doi: 10.3389/fimmu.2020.00929 (PMC7240028; doi:10.3389/fimmu.2020.00929)
Supplement: Supplementary file 1 [file Table_1.DOCX]

**Supplementary table 1. Number of available samples at each time point for PPD and ESAT6/CFP10 responses**

| **Time point** | **Responses to PPD** | | |  | **Responses to ESAT6/CFP10** | | |
| --- | --- | --- | --- | --- | --- | --- | --- |
| **(weeks)** | **LTBI-Negative (n=150)** | **LTBI-Positive (n=132)** | **Total (n=282)** |  | **LTBI-Negative (n=150)** | **LTBI-Positive (n=132)** | **Total (n=282)** |
| **0** | 138 | 116 | 254 |  | 72 | 78 | 150 |
| **1** | 71 | 58 | 129 |  | 41 | 37 | 78 |
| **4** | 66 | 53 | 119 |  | 38 | 39 | 77 |
| **6** | 57 | 56 | 113 |  | 38 | 41 | 79 |
| **10** | 57 | 51 | 108 |  | 41 | 39 | 80 |
| **14** | 21 | 32 | 53 |  | 21 | 32 | 53 |
| **24** | 27 | 29 | 56 |  | 27 | 29 | 56 |
| **52** | 110 | 95 | 205 |  | 109 | 95 | 204 |

*Table shows the number of samples assayed at each time point. Half of the infants were expected at time points other than weeks 0 and 52. Weeks 14 and 24 have lower numbers because they were introduced later.*
