# Supplementary material for: Maternal Latent Mycobacterium tuberculosis Does Not Affect the Infant Immune Response Following BCG at Birth: An Observational Longitudinal Study in Uganda
Source: Front Immunol. 2020 May 14;11:929. doi: 10.3389/fimmu.2020.00929 (PMC7240028; doi:10.3389/fimmu.2020.00929)
Supplement: Supplementary file 2 [file Table_2.DOCX]

**Supplementary table 2. Immune sensitisation based on cord blood responses to ESAT6/CFP10**

|  |  | **LTBI-Negative** | | |  | **LTBI-Positive** | |  |
| --- | --- | --- | --- | --- | --- | --- | --- | --- |
|  | **Cytokines** | **(n = 72)** | | |  | **(n = 78)** | | **p-value** |
| **Individual cytokines** | | |  |  |  | |  |  |
|  | IL2 | 3 | 4.2% | |  | 8 | 10.3% | 0.213 |
|  | IL5 | 1 | 1.4% | |  | 2 | 2.6% | 1.000 |
|  | IL10 | 60 | 83.3% | |  | 66 | 84.6% | 0.831 |
|  | IL13 | 19 | 26.4% | |  | 15 | 19.2% | 0.295 |
|  | IL17A | 6 | 8.3% | |  | 7 | 9.0% | 0.889 |
|  | TNF | 68 | 95.8% | |  | 72 | 94.7% | 1.000 |
|  | IFN-γ | 46 | 63.9% | |  | 44 | 56.4% | 0.350 |
| **Based on any of the 7 cytokines** | |  |  | |  |  |  |  |
|  | IL2, IL5, IL10, IL13, IL17A, TNF & IFN-γ | 71 | 98.6% | |  | 76 | 97.4% | 0.608 |
| **Based on PCA grouping** | | |  | |  |  |  |  |
|  | TNF & IL10 | 69 | 95.8% | |  | 75 | 96.2% | 1.000 |
|  | IL2, IL5, IL13 & IL17A | 22 | 30.6% | |  | 23 | 29.5% | 0.887 |
