# Supplementary material for: Maternal Latent Mycobacterium tuberculosis Does Not Affect the Infant Immune Response Following BCG at Birth: An Observational Longitudinal Study in Uganda
Source: Front Immunol. 2020 May 14;11:929. doi: 10.3389/fimmu.2020.00929 (PMC7240028; doi:10.3389/fimmu.2020.00929)
Supplement: Supplementary file 3 [file Table_3.DOCX]

**Supplementary table 3. Proportion of infants who developed cytokine responses to PPD at each time point**

|  | **Cytokines** | **LTBI-Negative** | |  | **LTBI-Positive** | | |
| --- | --- | --- | --- | --- | --- | --- | --- |
| **Week 1** | | **(n = 71)** | |  | **(n = 58)** | | |
|  | IL2 | 16 | 22.5% |  | 14 | 24.1% |  |
|  | IL5 | 44 | 62.0% |  | 33 | 56.9% |  |
|  | IL10 | 32 | 45.1% |  | 27 | 46.6% |  |
|  | IL13 | 51 | 71.8% |  | 47 | 81.0% |  |
|  | IL17A | 42 | 59.2% |  | 33 | 56.9% |  |
|  | TNF | 60 | 84.5% |  | 44 | 75.9% |  |
|  | IFN-γ | 37 | 52.1% |  | 31 | 53.4% |  |
| **Week 4** | | **(n = 66)** | |  | **(n = 53)** | | |
|  | IL2 | 38 | 57.6% |  | 37 | 69.8% |  |
|  | IL5 | 57 | 86.4% |  | 43 | 81.1% |  |
|  | IL10 | 23 | 34.8% |  | 28 | 52.8% |  |
|  | IL13 | 59 | 89.4% |  | 49 | 92.5% |  |
|  | IL17A | 59 | 89.4% |  | 48 | 90.6% |  |
|  | TNF | 60 | 90.9% |  | 50 | 94.3% |  |
|  | IFN-γ | 45 | 68.2% |  | 46 | 86.8% |  |
| **Week 6** | | **(n = 57)** | |  | **(n = 56)** | | |
|  | IL2 | 34 | 59.6% |  | 31 | 55.4% |  |
|  | IL5 | 52 | 91.2% |  | 50 | 89.3% |  |
|  | IL10 | 25 | 43.9% |  | 32 | 57.1% |  |
|  | IL13 | 53 | 93.0% |  | 50 | 89.3% |  |
|  | IL17A | 50 | 87.7% |  | 52 | 92.9% |  |
|  | TNF | 53 | 93.0% |  | 53 | 94.6% |  |
|  | IFN-γ | 48 | 84.2% |  | 50 | 89.3% |  |
| **Week 10** | | **(n = 57)** | |  | **(n = 51)** | | |
|  | IL2 | 43 | 75.4% |  | 34 | 66.7% |  |
|  | IL5 | 54 | 94.7% |  | 49 | 96.1% |  |
|  | IL10 | 19 | 33.3% |  | 22 | 43.1% |  |
|  | IL13 | 55 | 96.5% |  | 49 | 96.1% |  |
|  | IL17A | 51 | 89.5% |  | 48 | 94.1% |  |
|  | TNF | 54 | 94.7% |  | 49 | 96.1% |  |
|  | IFN-γ | 52 | 91.2% |  | 46 | 90.2% |  |
| **Week 14** | | **(n = 21)** | |  | **(n = 32)** | | |
|  | IL2 | 14 | 66.7% |  | 24 | 75.0% |  |
|  | IL5 | 19 | 90.5% |  | 30 | 93.8% |  |
|  | IL10 | 16 | 76.2% |  | 25 | 78.1% |  |
|  | IL13 | 20 | 95.2% |  | 32 | 100.0% |  |
|  | IL17A | 19 | 90.5% |  | 29 | 90.6% |  |
|  | TNF | 20 | 95.2% |  | 31 | 96.9% |  |
|  | IFN-γ | 20 | 95.2% |  | 32 | 100.0% |  |
| **Week 24** | | **(n = 27)** | |  | **(n = 29)** | | |
|  | IL2 | 24 | 88.9% |  | 25 | 86.2% |  |
|  | IL5 | 26 | 96.3% |  | 28 | 96.6% |  |
|  | IL10 | 20 | 74.1% |  | 19 | 65.5% |  |
|  | IL13 | 26 | 96.3% |  | 29 | 100.0% |  |
|  | IL17A | 26 | 96.3% |  | 27 | 93.1% |  |
|  | TNF | 27 | 100.0% |  | 29 | 100.0% |  |
|  | IFN-γ | 26 | 96.3% |  | 29 | 100.0% |  |
| **Week 52** | | **(n = 110)** | |  | **(n = 95)** | | |
|  | IL2 | 92 | 83.6% |  | 84 | 88.4% |  |
|  | IL5 | 92 | 83.6% |  | 78 | 82.1% |  |
|  | IL10 | 66 | 60.0% |  | 59 | 62.1% |  |
|  | IL13 | 102 | 92.7% |  | 87 | 91.6% |  |
|  | IL17A | 91 | 82.7% |  | 80 | 84.2% |  |
|  | TNF | 106 | 96.4% |  | 91 | 95.8% |  |
|  | IFN-γ | 101 | 91.8% |  | 89 | 93.7% |  |
