# Supplementary figures and images for: Maternal Latent Mycobacterium tuberculosis Does Not Affect the Infant Immune Response Following BCG at Birth: An Observational Longitudinal Study in Uganda
Source: Front Immunol. 2020 May 14;11:929. doi: 10.3389/fimmu.2020.00929 (PMC7240028; doi:10.3389/fimmu.2020.00929)

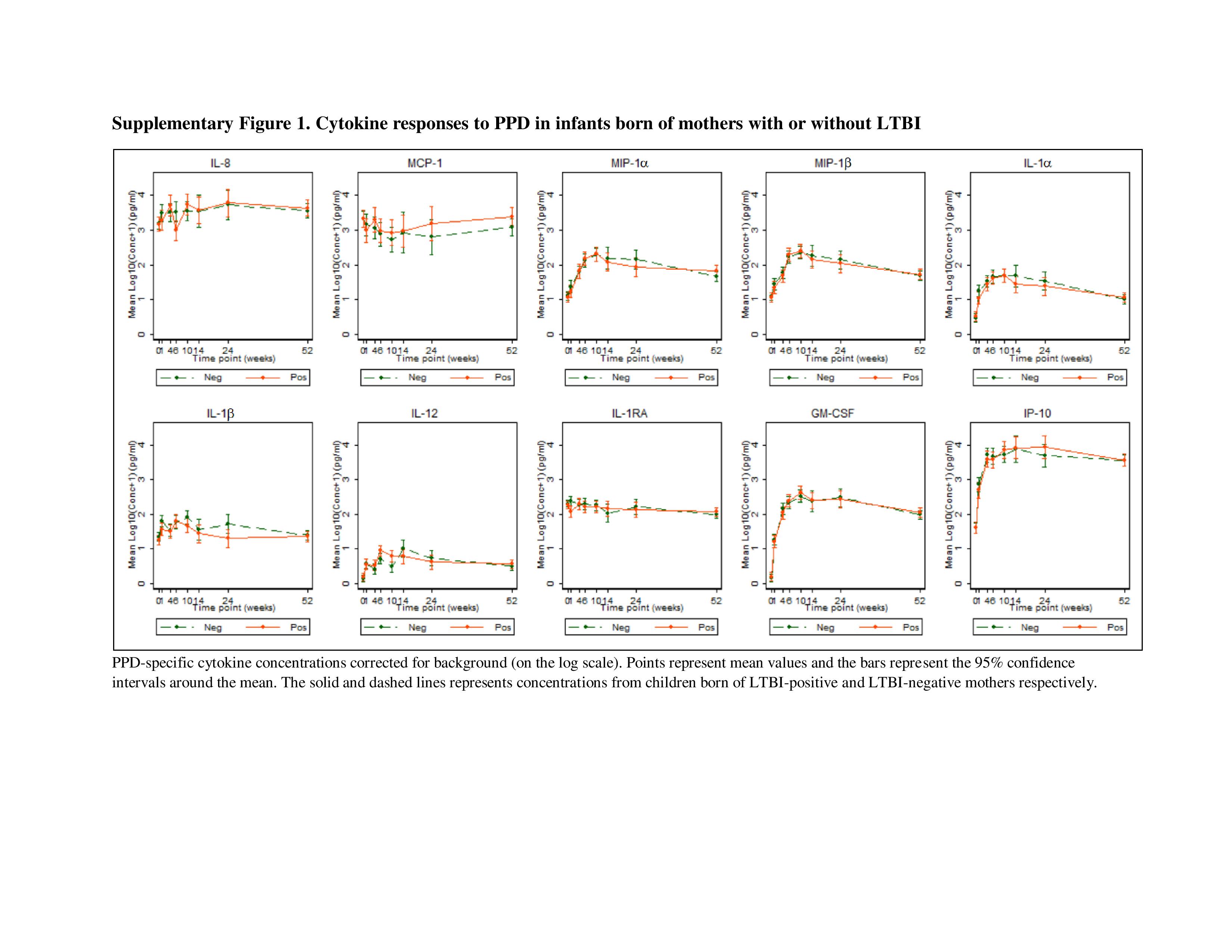

Supplement: Supplementary file 4 [file Image_1.JPEG]

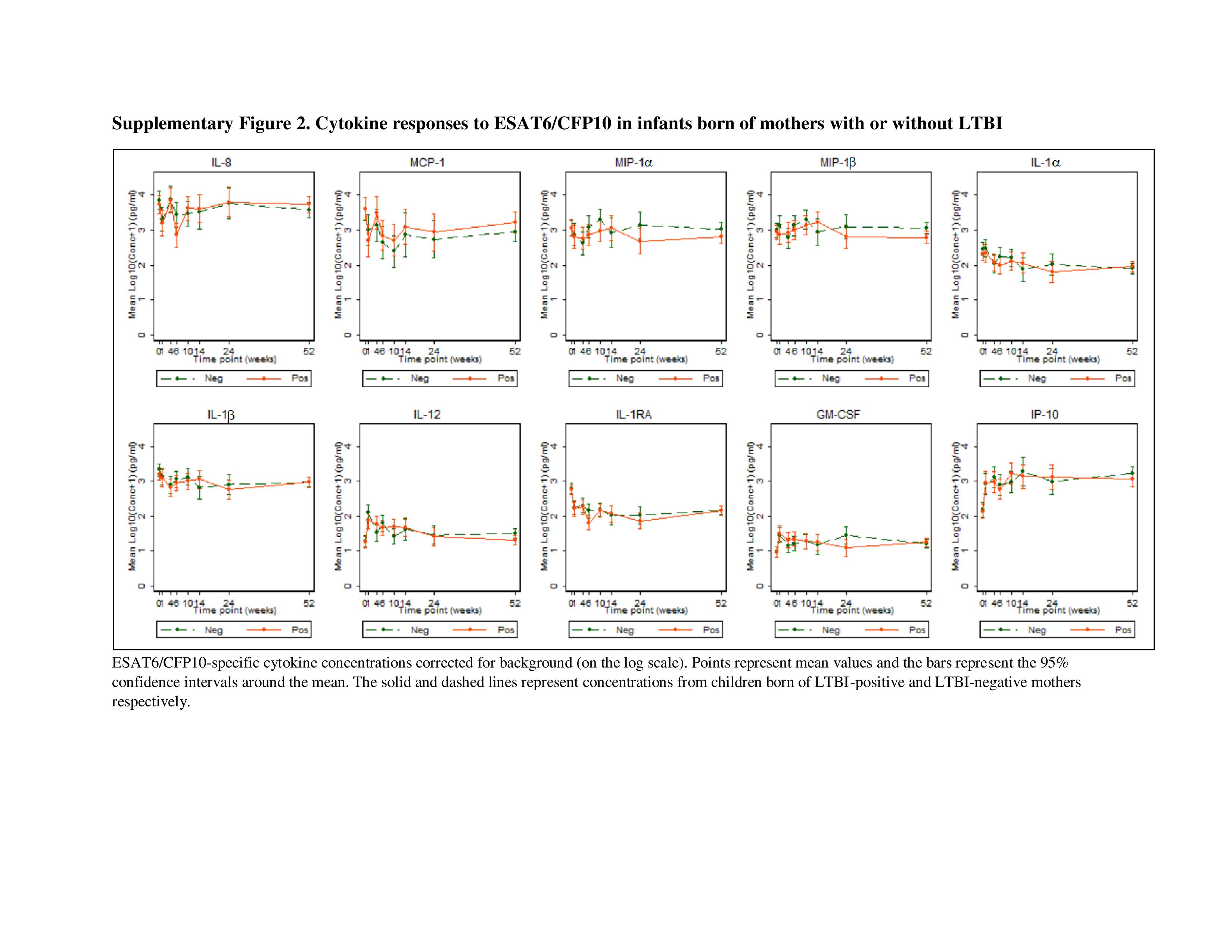

Supplement: Supplementary file 5 [file Image_2.JPEG]

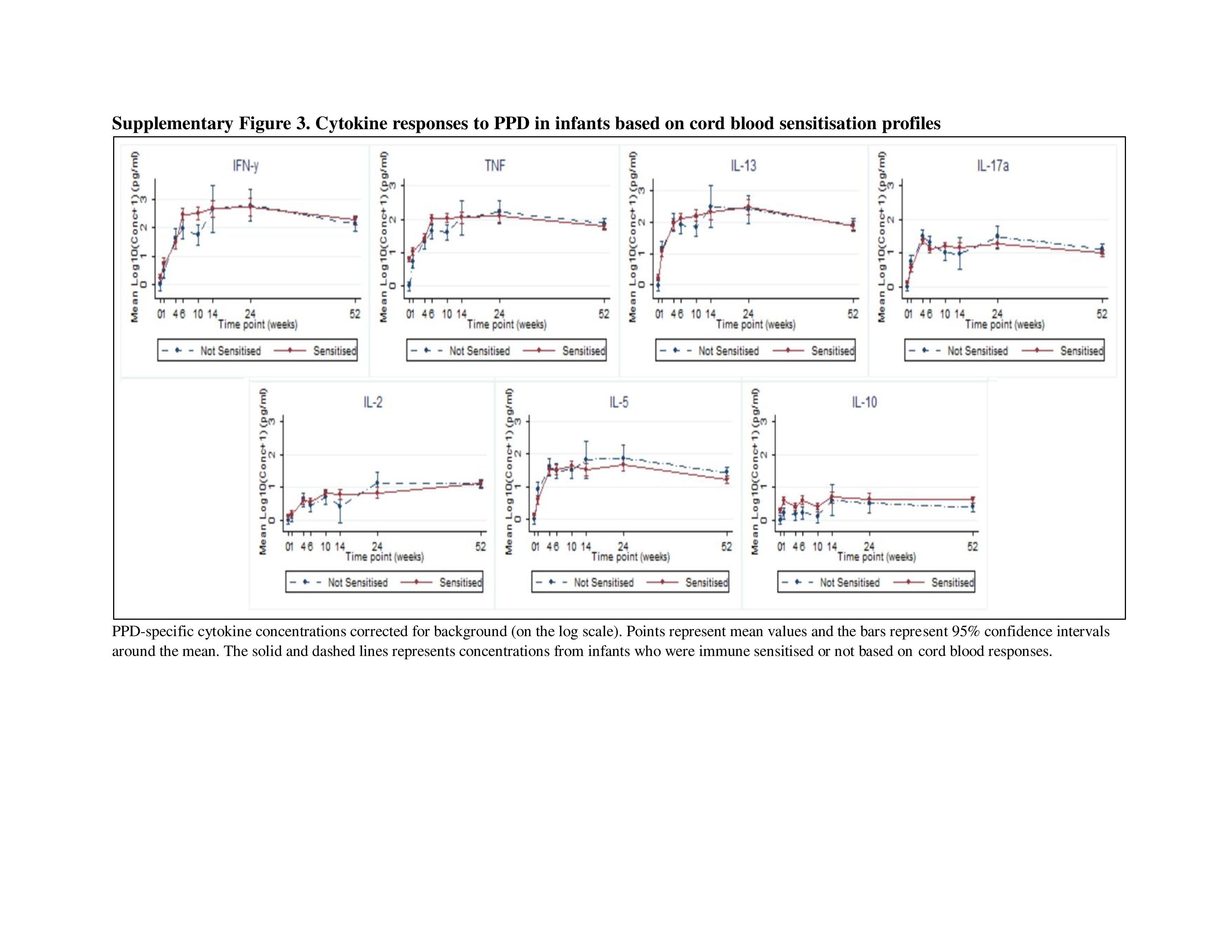

Supplement: Supplementary file 6 [file Image_3.JPEG]

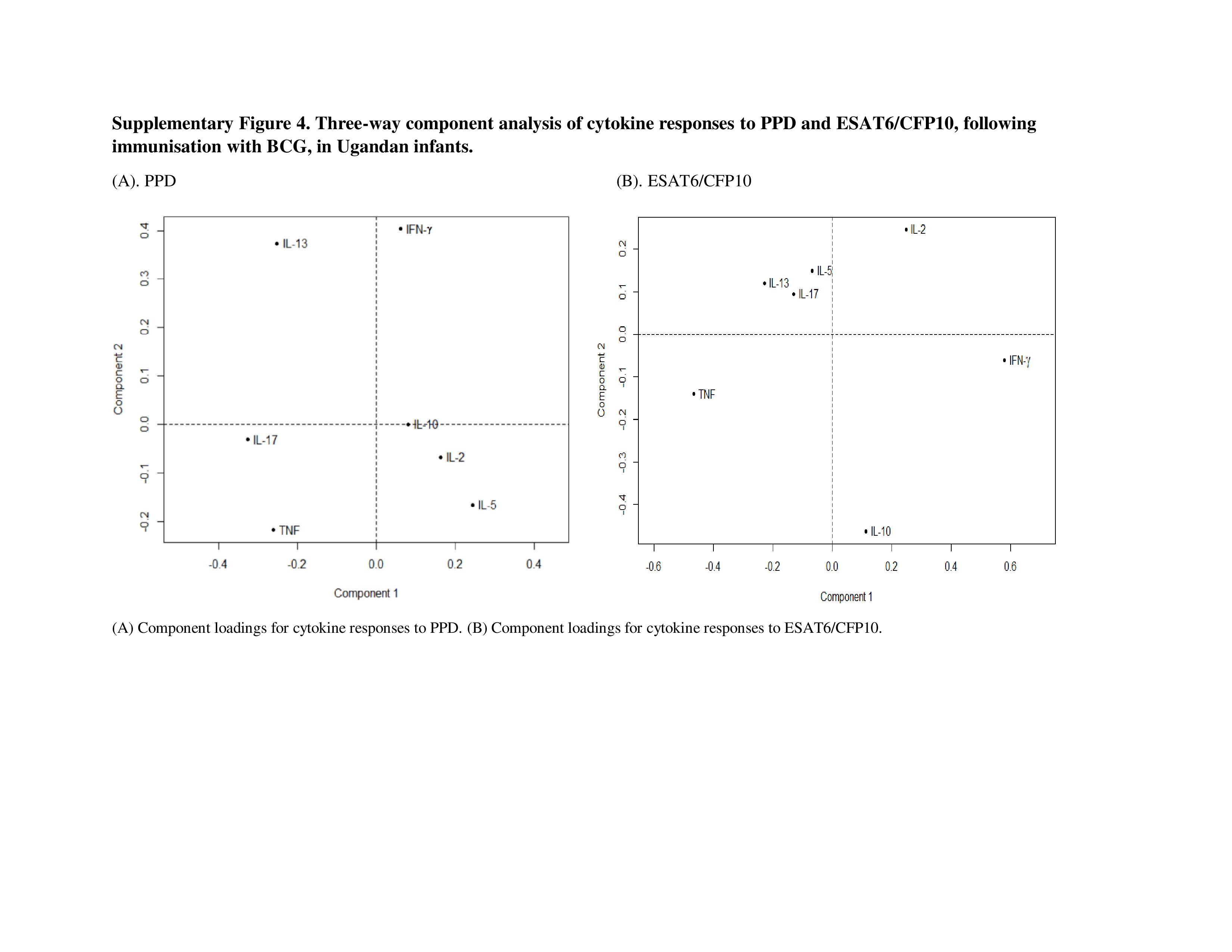

Supplement: Supplementary file 7 [file Image_4.JPEG]
